# Work capacity and health-related quality of life among individuals with multiple sclerosis reduced by fatigue: a cross-sectional study

**DOI:** 10.1186/1471-2458-13-224

**Published:** 2013-03-15

**Authors:** Gullvi Flensner, Anne-Marie Landtblom, Olle Söderhamn, Anna-Christina Ek

**Affiliations:** 1 Department of Medicine and Health, Division of Nursing Science, Faculty of Health Sciences, Linköping University, Linköping, SE-581 85, Sweden; 2 Department of Nursing, Health and Culture, University West, Trollhättan, SE-461 86, Sweden; 3 Department of Clinical and Experimental Medicine (IKE), Division of Neurology, Faculty of Health Sciences, Linköping University, UHL and LiM, County Council of Östergötland, Linköping, SE-581 83, Sweden; 4 Centre for Caring Research-Southern Norway, Faculty of Health and Sport Sciences, University of Agder, PO Box 509, Grimstad, NO-4898, Norway

**Keywords:** Cognition, Education, Emotional distress, Heat sensitivity, Regression analysis

## Abstract

**Background:**

Among individuals diagnosed with the chronic neurologic disease, multiple sclerosis (MS), a majority suffers from fatigue, which strongly influences their every-day-life. The aim of this study was to investigate work capacity and health-related quality of life (HRQoL) in a group of MS patients and also to investigate if work capacity and HRQoL could be predicted by background factors, fatigue, heat sensitivity, cognitive dysfunction, emotional distress or degree of disability.

**Methods:**

A descriptive, cross-sectional, designed survey was undertaken A questionnaire was sent to 323 individuals diagnosed with MS, aged between 20 and 65 years, with physical disability on the expanded disability status score (EDSS) in the interval 0 ≥ EDSS ≤ 6.5, living in Östergötland county in eastern Sweden. Questions on background factors, occupation and work, together with the health-related quality of life short form instrument (SF-36), the fatigue severity scale (FSS), the perceived deficit questionnaire (PDQ) and the hospital anxiety depression scale (HAD) were posed. Associations between variables were analyzed using Pearson’s and Spearman’s correlations. Differences between groups were tested using the Chi-square test, the Mann Whitney *U*-test, and the Student’s *t*-test. Predictive factors were analyzed using multiple linear and multiple logistic regression analysis.

**Results:**

Of those who completed the questionnaire (n = 257, 79.6%), 59.8% were working. Work capacity was found significantly more among men (p < 0.005), those with a higher level of education (p < 0.001), those reporting less fatigue (p < 0.001), and those having no heat sensitivity (p = 0.004). For work capacity, significant predictors were low physical disability (EDSS), low fatigue, higher level of education, male sex and lower age. Those with work capacity showed significantly higher HRQoL than those who had no work capacity (p < 0.001). Levels of fatigue, cognition and emotional distress were found to be major contributing factors for HRQoL.

**Conclusions:**

Work capacity and HRQoL among individuals diagnosed with MS are highly influenced by fatigue which can be considered as a key symptom. Work capacity was influenced by heat-sensitivity, cognitive difficulties and emotional distress and significant predictive factors besides fatigue, were physical disability (EDSS), age, sex, and level of education. Remaining at work also gives a better HRQoL.

## Background

Among people visiting general practitioners, fatigue is the major reason for the visit in five to seven percent [1], and among people in the working population, 21 - 23% have been found to suffer from fatigue [2,3]. There is no single explanation for what causes fatigue, but it often accompanies chronic diseases [4-6].

Among individuals with the chronic neurologic disease, multiple sclerosis (MS), the majority, 60-80%, report that they suffer from fatigue [3,7]. As many as one third of MS-patients noted fatigue as their very first symptom and they noted it before other neurological signs of the disease became apparent [8]. The experience of MS-related fatigue is described as quite distinguished from any prior experiences of feeling tired [9]. Fatigue related to MS is strongly influencing the individuals’ daily lives [3,5,10], and reduces their quality of life [11,12].

Onset of MS often happens in early adulthood, 70% in 20–40 years of age [13] and life expectancy is long, about 65 years of age [14], which means that many people in the Western world are affected. For example, in European countries there are about half a million people diagnosed with MS [15]. In Sweden, prevalence studies show that about 180/100 000 inhabitants have been diagnosed with MS [16,17]. To a large extent, MS is a female problem and women get MS about twice as often as men; in Sweden, recently reported sex ratios are 2.65 and 2.35 (female/male) [16,18]. In about 70-80% of cases, the initial course is relapsing-remitting (RR) [13], however, after an average of 15 years and in about 70% of cases, the RR course changes to a secondary progressive course (SP) characterized by increasing disability. In 20%, the course is progressive from the onset, called primary progressive course (PP) [13]. Among young and middle aged adults, MS is the main reason for disability and limitations in functional abilities [19]. The young age of onset in MS makes it one of the major causes of reduced work capacity due to neurologic disease in Western society. Importantly, of all its symptoms, a majority of the individuals with MS consider fatigue as the most handicapping and as their worst problem [3]. Individuals with a RR-course of the disease have, in a large sample and a longitudinal study, been found to have less severe fatigue compared to individuals with a SP or PP-course [20].

Besides fatigue, individuals with MS may experience other problems influencing their work capacity and quality of life. Cognitive difficulties such as memory and attention problems [9,21] are found as affecting about half of the individuals with MS and described as a leading cause of perceived disability in MS [22,23]. Among a group of young MS-patients [24], one third showed cognitive impairment in complex attention, i.e. in rapid shifting between competing stimuli, which was assumed to affect their work and career [24].

However, there have also been reported differences between self- graded cognitive function and observed neuropsychological performance [25].

Another well-known feature of fatigue in MS is its association with depression and over 50% experience emotional distress, a symptom intertwined with the experiences of fatigue and health related quality of life [26,27]. Subsequently, several frequent MS symptoms appear concurrently: fatigue, depression, cognitive dysfunction and, in addition to these, heat sensitivity which has been increasingly focused upon [28-31]. Heat sensitivity has been shown to increase fatigue and other common MS-symptoms, like pain and difficulties concentrating [31-33], all of which may influence individuals’ disability and work capacity.

Reduced capacity to work is a major cause of the high cost of MS in society, as are the costs associated with the need for personal care in later stages of the disease. One study showed that the cost of MS in Sweden is about €600 million a year, one third of which are indirect costs associated with loss of production [34]. In a Danish study [35], people with MS left paid work earlier than people in the general population. After five years, 30% of MS patients (n = 2 538) had retired early, compared to 3% in the control group and twenty years later the proportions were 78% compared to 14% respectively. In a Swedish study from the early 1990s, about 50% of those with MS were on full sick leave [36], compared to 37.5% in a recent study [37]. The earlier studies [35,36] were performed before immune-modulating drugs were introduced and currently many patients with MS are treated with such drugs, the effects of which have been focused on in relation to both fatigue and work ability. These drugs may decrease the development of disability [38] and increase work ability [37], but they may also increase fatigue [39,40].

This study is part of a wider project on the topic of MS fatigue carried out in Östergötland county in eastern Sweden by our research group [3,31,41]. In this study, work capacity and health related quality of life (HRQoL) among individuals diagnosed with MS aged 65 or younger were investigated and, further, whether work status and HRQoL can be predicted by background factors, fatigue, heat sensitivity, cognitive dysfunction, emotional distress or degree of disability were analyzed.

## Methods

### Study group

A descriptive, cross-sectional, designed survey was undertaken. Recruitment to the study was done among people living in Östergötland county in eastern Sweden who were registered in the Swedish MS register (SMS register). The following criteria for participation were established: i) diagnosed with MS, ii) of working age, i.e. between 20 and 65 years, 65 years is the official retirement age in Sweden, and iii) having an Expanded Disability Status Score (EDSS) [42] in the interval 0 ≥ EDSS ≤ 6.5. Using EDSS, an individual’s physical disability is measured based on ratings of neurological signs of neurological functions and ambulation, graded in twenty steps ranging from zero to ten. An EDSS = 0 indicates normal neurological conditions, while EDSS = 10 indicates the individual’s death due to MS. In this study, the chosen interval of EDSS indicates that most of the individuals are mobile and can walk at least short distances [42].

A package containing an information letter about the study, a questionnaire and a pre-paid reply envelope was sent to each of the 323 individuals fulfilling the criteria, 239 women and 84 men. The questionnaires were distributed in 2007 and 2008. One reminder was sent to those who had not answered within three weeks. Other analyses and results have been published from this study [31].

### Data collection

Information about the participants’ age and sex, disease course, actual disability assessed with EDSS [42] and date of onset was acquired from the SMS register. Both the patient’s disease-course and EDSS are continuously upgraded when visiting a physician at the neurological policlinic. The participants were grouped according to normal neurological condition (EDSS = 0), mild disability (1.0 ≥ EDSS ≤ 3.5), moderate disability (4.0 ≥ EDSS ≤ 5.5) and severe disability (6.0 ≥ EDSS ≤ 6.5).

Information on background factors such as the participants’ civil status, family, level of education together with on-going medical treatment was requested in the questionnaire. In one question, the participants’ occupation and to what degree they worked, were requested. The participants specified if they were working full-time, part-time or not working. In the case of part-time work, the participants reported percentages of full-time. They also reported if they were on full- or part-time sick leave or received a disability pension. Ongoing education and training and working at home were considered as equivalent to work. In one question, the participants were asked “Are you sensitive to heat?” (Yes/No).

HRQoL was assessed using the Swedish version of the well-tested and extensively used generic instrument short form SF-36 Health Survey [43-45]. The questionnaire measures eight health domains (subscales): physical functioning (PF), role-physical (RP), bodily pain (BP), general health (GH), vitality (VT), social functioning (SF), role-emotional (RE), and mental health (MH). Each subscale contains 2 to 10 items, for a total of 35 questions. In an additional item, the respondents rated their current health status compared to one year previously on a five-point scale (1 = much better to 5 = much worse). Subscale scores are computed according to standardized procedures and range from 0 to 100, in which higher scores indicate better health related quality of life [43,46].

To investigate the perceived severity of fatigue, the Fatigue Severity Scale (FSS) [47] was used, comprising nine items with each item graded from least fatigue (1) to severe fatigue (7). As in earlier studies [e.g. 7, 48], an FSS mean score ≤4 was regarded as indicating no fatigue, >4 but <5 indicated borderline fatigue, and ≥5 indicated severe fatigue. The FSS is widely used both clinically and in research [48]. In this study, concurrent validity was assessed through correlations between the FSS total with questions about the impact of fatigue on daily life and the impact on the ability to work, and resulted in *r* =0.79 (*p* < 0.01) and *r* =0.75 (*p* < 0.01) respectively. Reliability was assessed using Cronbach’s alpha [49], and the alpha coefficient was 0.93 [31].

To investigate perceived problems with memory, attention and concentration, the Perceived Deficit Questionnaire (PDQ) [50] was translated from the original English into Swedish. The Swedish version was back-translated in accordance with Streiner and Norman [51]. In PDQ, 20 items are graded from never (0) to always (4), and summated scores vary from 0 to 80, higher scores indicate greater cognitive problems. Concurrent validity was estimated by calculating correlations between the PDQ sum score and the graded occurrence of two MS symptoms, forgetfulness and difficulties concentrating (never = 0 to always = 5) and resulted in r = 0.75 (p < 0.010) and r = 0.73 (p < 0.010) respectively. These results indicate that there is evidence of concurrent validity for PDQ in this study group. Reliability was tested using Cronbach’s alpha, the alpha coefficient was 0.95, and the split-half technique (r = 0.87).

To investigate the occurrence of emotional distress, the widely used Hospital Anxiety Depression (HAD) scale was applied [52]. In seven items, occurrence of symptoms mirroring anxiety and depressive symptoms respectively are graded from zero to three. Summated scores range from 0 to 21 and identify non-cases (score 7 or less), doubtful cases (scores in the interval 8–10) and definitive cases (scores 11 or higher). In this study, reliability assessed using Cronbach’s alpha, resulted in the alpha coefficient for anxiety being 0.88 and for depression 0.87.

### Statistical analyses

The descriptive statistics used are numbers and per cents, median and interquartile-range (IQR) mean and standard deviation (SD). The scales FSS, PDQ and SF-36 are treated on interval levels as in earlier studies. Associations between variables were tested by Pearson’s or Spearman’s correlation calculations. Differences between groups were tested with the Chi-square test on data on a nominal level, with the Mann Whitney *U*-test on data on an ordinal level, and Student’s *t*-test on data on an interval level. In the multiple logistic regression analyses (enter), the capacity for work, dichotomised as capacity (full or limited = 1) or no capacity (full-time sick leave or disability pension = 0) was entered as a dependent variable, and fatigue (FSS-mean), heat sensitivity, cognition, emotional distress, disability, age, gender (female = 1/male = 2), and level of education (compulsory = 1, secondary school or university = 2) as independent variables. The correlation between age and having children was r_s_ = 0.78, therefore, age was used in the logistic analyses model. In the multiple linear regression analyses (enter) the domains in HRQoL measured by SF-36 were used as dependent variables, and fatigue, heat sensitivity, cognition, anxiety, depression, disability, age and gender were used as independent variables.

### Ethical considerations

Common ethical principals in research, and in accordance with the Declaration of Helsinki [53], were applied in the study. Approval to use the SMS register was received from the local administrator responsible for the register. The Regional Ethical Review Board at the Faculty of Health Sciences, Linköping University, Sweden (Dnr M13-07) also approved the study. A completed and returned questionnaire was considered received informed consent.

## Results

### Participants

Two hundred and fifty seven participants (79.6%) responded to the invitation and returned a completed questionnaire; of the surveyed, 76.3% were women and 23.7% were men. There were no significant differences between non-respondents and respondents regarding sex (*p* = 0.066), disease-course (*p* = 0.064) or EDSS score (*p* = 0.142). The participants’ mean age was 47.5 years (SD10.8), the women (n = 196) were older (48.2 years, SD 11.0) than the men (n = 61), (44.8 years, SD10.1) (*p* = 0.033) (Table 1), while the non-respondents were five years younger on average, m = 42.7 years (SD 10.5) (*p* < 0.001).

**Table 1:** Sample characteristics

	**Women (n = 196)**	**Men (n = 61)**	**P-value**
*Age*, years Mean (SD)	48.2 (11)	44.8 (10)	0.033
*Civil status*, n (%)			
Married/cohabitant, n	141(72)	44 (72)	0.959
Living alone, n	55 (28)	17 (28)	
*Education*, n (%)			
Lower: Compulsory – Secondary school	123 (63)	45 (70)	0.125
Higher: After secondary school – University	72 (37)	16 (30)	
Missing data	1		
*Occupation*			
Working, full- or part-time, n (%)	106 (54)	47 (77)	0.005
Not working, n (%)	89 (46)	14 (23)	
Missing data	1		
*Course of the disease*, n (%)			
Relapsing – remitting (RR)	140 (71)	47 (77)	0.389
Progressive (PP + SP)	56 (29)	14 (23)	
*Disease severity*, n (%)			
No neurological signs, EDSS = 0	20 (10)	7 (11)	0.560
Mild, 1.0 ≤ EDSS ≤ 3.5	119 (61)	38 (62)	
Moderate, 4.0 ≤ EDSS ≤ 5.5	21 (11)	9 (15)	
Severe, 6.0 ≤ EDSS ≤ 6.5	36 (18)	7 (11)	
*Medication*			
No pharmacological treatment, n (%)	45 (23)	9 (15)	0.164
Pharmacological treatment, n (%)	150 (77)	52 (85)	
*Immune-modulating treatment*			
Yes, n (%)	103 (53)	41 (67)	0.828
No, n (%)	93 (47)	20 (33)	

Characteristics of participants (n = 257).

### Work capacity

Of the respondents, 59.8% reported that they worked full- or part-time. Regarding background factors in relation to work capacity, significantly more women than men were on full-time sick leave or disability pension (Table 1). Those participants who had capacity to work were younger, mean age 44.5 years (SD10.4) compared to those who had non-capacity to work, mean age 51.8 years (SD10.2) (*p* < 0.001). Besides disability, significant differences between the levels of capacity to work were found, for example, regarding fatigue, heat sensitivity, cognitive difficulties and emotional distress (Table 2).

**Table 2:** Participants in relation to capacity to work or non capacity to work

	**Capacity to work (n = 153)**	**Non capacity to work (n = 103)**	**P-value**
*Fatigue, FSS, range 1-7*			
- Mean (SD)	4.2 (1.6)	5.3 (1.4)	< 0.001
*Heat- sensitivity*, n			
- Yes, n = 148 (%)	77 (52)	71 (48)	
- No, n = 108 (%)	76 (70)	32 (30)	0.004
*Cognitive dysfunction, PD*Q, range, 0-80			
- Mean (SD)	24.2 (13.5)	30.0 (16.2)	0.002
*Education*			
- Compulsory and secondary school, n (%)	86 (57)	81 (79)	
- University, n (%)	66 (43)	22 (21)	< 0.001
*Emotional distress, HAD*,			
- Anxiety (HAD), range 0–21, Md (IQR)	3 (6)	6 (7)	0.303
- Depressed (HAD), range 0–21, Md (IQR)	3 (4)	4 (6)	0.002
*Disability*			
- Normal neurological status (EDSS = 0), n (%)	25 (92)	2 (8)	
- Mild disability (EDSS 1.0 – 3.5), n (%)	113 (72)	44 (28)	< 0.001
- Moderate disability (EDSS 4.0 – 5.5), n (%)	9 (31)	20 (69)	
- Severe disability (EDSS 6.0-6.5), n (%)	6 (14)	37 (86)	
*Medication*			
- No pharmacological treatment, n (%)	23 (15)	30 (29)	0.007
- Pharmacological treatment, n (%)	129 (85)	73 (71)	
*Immune-modulating treatment*			
- Yes, n (%)	109 (71)	36 (35)	< 0.001
- No, n (%)	44 (29)	63 (65)	

Capacity to work (full- or part-time) or non capacity to work described in relation to fatigue (FSS), heat-sensitivity, cognitive dysfunction (PDQ), educational level, anxiety and emotional distress (HAD), and disability (EDSS).

*FSS* = Fatigue Severity Scale;

*PDQ* = Perceived Disability Questionnaire;

*HAD* = Hospital Anxiety Depression Scale;

*EDSS* = Expanded Disability Status Scale;

Missing value, n = 1.

Fatigue (FSS-mean) correlated significantly with cognitive difficulties (PDQ-sum), r = 0.51 (p < 0.001). The participants who reported that they were sensitive to heat (n = 146) also reported significantly more cognitive difficulties compared to those who were not sensitive to heat (n = 108), mean 29.5 (SD14.8) and 22.6 (SD14.4), (p < 0.001), respectively.

Seventy five percent of the participants with a university education (n = 88) reported that they were working, compared to 51.5% of the participants with compulsory or secondary school education (n = 167) (p < 0.001). The latter graded significantly more cognitive difficulties than the participants with a university education, mean 29.3 (SD15.3) and 21.6 (SD12.9), (p < 0.001), respectively.

In a multiple logistic regression analysis, (enter), 54% of the capacity for work was explained by the model. Significant factors in the model were fatigue (FSS-mean) (OR = 0.75, p = 0.041), disability assessed by EDSS (OR = 0.53, p <0.001), sex (OR = 4.34, p = 0.001), level of education (OR = 3.88, p = 0.002) and age (OR = 0.93, p <0.001) (Table 3).

**Table 3:** Logistic regression analyses of capacity for work

**Dependent variable**	**Predictors**	**OR**	**95%Cl**	**P-value**
*Capacity for work*	Fatigue (FSS-mean)	0.75	0.57-0.99	0.041
	Disability (EDSS) (0–6.5)	0.53	0.43-0.65	<0.001
	Sex (male = 2)	4.34	1.76-10.67	0.001
	Educational level	3.9	1.6-9.1	0.002
	Age	0.93	0.90-0.97	<0.001

The significant factors in the logistic regression analyses of capacity for work, dichotomized as full or limited = 1 or non capacity = 0 as a dependent variable, and fatigue, heat sensitivity, cognition, anxiety, depression, disability, gender, educational level and age as independent variables.

R^2^ Nagelkerke = 0.54.

### Health-related quality of life

Fatigue significantly influenced daily life (p < 0.001). Among those participants who reported that they were sensitive to heat, self-rated health (first item in the SF-36) was rated significantly worse (mean 3.5, SD 0.83) compared to those who were not sensitive to heat (mean 3.2, SD 0.92) (*p* = 0.015). HRQoL was graded significantly higher in all SF-36 domains among those who were working full- or part-time compared to those who did not work (Table 4).

**Table 4:** Health-related quality of life

	**Capacity to work**	**Non capacity to work**	**P-value**
*Health-related Quality of life* (SF-36)			
- Physical functioning, mean (SD)	75.5 (22.0)	41.7 (26.4)	< 0.001
- Role functioning, mean (SD)	55.0 (39.5)	25.8 (36.3)	< 0.001
- Body pain, mean (SD)	71.1 (27.2)	53.4 (29.7)	< 0.001
- General health, mean (SD)	56.8 (22.3)	42.7 (18.9)	< 0.001
- Vitality, mean (SD)	50.9 (22.5)	38.6 (23.5)	< 0.001
- Social functioning, mean (SD)	71.2 (25.5)	59.3 (26.3)	< 0.001
- Role emotional, mean (SD)	72.0 (37.9)	57.0 (45.7)	0.005
- Mental health, mean (SD)	73.1 (20.3)	64.7 (20.6)	0.002

Health-related quality of life (HRQoL) (SF-36) in relation to capacity to work (full or part-time) or non capacity to work.

In the multiple linear regression analyses, fatigue was significantly related to HRQoL in all SF-36 domains except role-emotional and mental health (Table 5).

**Table 5:** Multiple regression analyses of the domains in health-related quality of life

**Dependent domains SF-36**	**Significant independent variables**	**Standardized ß**	**P-value**	**Adjusted R**^**2**^**in the model**
*Physical functioning* (PF)	Fatigue (FSS)	-.240	<0.001	0.57
	Disability (EDSS)	-.594	<0.001	
	Age	-.120	0.008	
*Role physical* (RP)	Fatigue (FSS)	-.451	<0.001	0.39
	Depression (HAD-D)	-.201	0.009	
	Gender	.118	0.026	
*Bodily pain* (BP)	Fatigue (FSS)	-.197	0.004	0.31
	Cognition(PDQ)	-.159	0.042	
	Anxiety – HAD-A	-.145	0.052	
	Depression – HAD-D	-.154	0.055	
	Age	-.134	0.019	
*General health* (GH)	Fatigue (FSS)	-.322	<0.001	0.35
	Anxiety (HAD-A)	-.164	0.028	
	Age	-.136	0.016	
*Vitality* (VT)	Fatigue(FSS)	-.562	<0.001	0.60
	Cognition (PDQ)	-.123	0.038	
	Depression(HAD-D)	-.182	0.003	
*Social functioning* (SF)	Fatigue (FSS)	-.387	<0.001	0.42
	Depression (HAD-D)	-.299	<0.001	
*Role emotional* (RE)	Heat sensitivity	-.107	0.054	0.39
	Anxiety (HAD-A)	-.234	<0.001	
	Depression (HAD-D)	-.322	<0.001	
*Mental health* (MH)	Anxiety(HAD-A)	-.447	<0.001	0.60
	Depression (HAD-D)	-.376	<0.001	

Multiple linear regression analyses of the domains in health-related quality of life measured by SF-36 and used as a dependent variable and fatigue, heat sensitivity, cognition, anxiety, depression, disability, age and gender as independent variables.

## Discussion

This study is a cross-sectional study and a questionnaire was used to collect the data. The response rate, nearly 80%, is consistent with what is common in similar studies. In this study, the women were somewhat older than the men and were also more often retired due to the disease, while more men reported they had work capacity. Regarding civil status, level of education, course of disease and disability or pharmacological treatment, there were no significant differences between women and men, except for work capacity.

Fatigue proved to be a significant factor influencing work capacity. Those participants who had the capacity to work reported significantly less fatigue compared to those with no capacity to work. Another fact was that the level of work capacity was statistically significantly higher among those participants who were not sensitive to heat, while those who were sensitive to heat showed significantly more often a non-capacity to work. In a previous study, heat sensitivity was found to significantly interfere with and also increase fatigue [31]. In this study, fatigue could be seen as a key symptom influencing both work capacity and HRQoL.

Similar to the findings in this study, Krokavcova et al. [54] found that patients with self-rated good health are more likely to be employed. In this study, participants who had the capacity to work graded both better health and HRQoL than those with a non-capacity to work.

Interestingly, a higher level of education was shown as a significant predictive factor for work capacity in this study. The reason for this may be discussed in terms of disability level and work of a person, which demands that the person is movable, or that educational attainment has not been disrupted due to MS. This might be dependent of the age at onset of the disease [24]. In the present study, just a few percent of the participants (5.5%) had onset of the disease before 20 years of age [31]. Blue collar work usually consists of physical labor which naturally is influenced by actual disability, while work requiring higher education might more often be paperwork or white collar work. This result indicates the importance of supporting young MS-patients in continuing their education, even at a higher level. This would also be in line with the results presented by Glad and colleagues [26], who found that having a RR-course, higher education and light physical work were predicting factors for being able to remain employed.

Another interesting aspect of the increased work capacity of patients with higher education is that cognitive problems in MS are a common feature. However, obviously, in this study, such problems did not interfere with work, probably based on intellectual processes, which required higher education. However, MS is a complex disease and regarding fatigue, multiple factors might interact and influence both fatigue and cognitive function. In this study in the linear regression analysis, fatigue and cognition were found as contributing factors in the domain of bodily pain and vitality. Pain may interfere with and disrupt an individual’s attention and concentration, but also influence fatigue. An important question is if cognitive problems in MS sometimes fluctuate and are secondary to fatigue. This should be of interest to study further. However, fluctuations of cognitive difficulties in MS have been found in relation to a heat environment [31-33]. In one study, cognitive difficulties were reported as eased when the individual used a cooling-suit [29], which also should be of interest for further investigations.

Age appeared as a predictor for capacity for work. In a correlation analysis we found that age and having children were closely correlated (r_s_ = 0.78) why we chose to keep age as the independent variable. In our original hypothesis, however, we paid an interest in MS patients with small children since this situation may increase their level of fatigue. As age appeared as a predictor we also suggest that it stands for having small children.

Quality of life was assessed as significantly higher among those who worked, a result also found in earlier studies. Obvious in this study was that fatigue was a significant factor influencing all domains of health-related quality of life, except role-emotional and mental health, while cognition was a significant factor influencing two of the domains, bodily pain and vitality. In the domain of role-emotional, heat sensitivity appeared as a significant influencing factor which might be understood as limiting the individuals’ social life activities.

The impact of fatigue on quality of life is obviously connected to work ability, a central function in everybody’s life. For natural reasons, the risk of isolation in society increases both through unemployment and the obstacles it places to participating in family life on equal terms. In conclusion, the impact on these domains could very well affect the individual’s sense of coherence which is central to the individual’s perception of wellbeing. As fatigue is a symptom with a severe impact on quality of life and work capacity, it is important to find the organic cause which, in the case of MS, justifies a patient’s sick leave and disability pension.

Another point of view is that being able to work is of importance for the quality of life of people with chronic diseases. People with MS, should have the opportunity to work part-time as long as possible. As people with MS are often affected early on in life, their occupational development is more difficult. The fact that young people may be stopped by MS from working at all should also be taken into account.

Apart from disability measured by EDSS, the fact that heat sensitivity interferes with work capacity suggests that organising cryotherapeutic approaches in the work place, such as air conditioning, cooling garments and disability friendly measures should be put in place.

This article also focuses on fatigue as a common and serious symptom in MS already at the onset and the following stages of the disease. For example, general practitioners should take MS-fatigue into consideration when young patients complain about tiredness. Subsequently, it is necessary that patients are properly diagnosed early on in the course of a disease like MS, indicating that physicians should be careful when diagnosing a common symptom like tiredness or mental/physical exhaustion in young people, especially females. However, all patients who complain of fatigue cannot be referred to a neurologist. The Swedish sick leave system relies on patients being diagnosed. Clear recommendations are made in order to determine the appropriate sick leave period with a specific diagnosis. This means that a patient without a specific diagnosis, i.e. a rather symptomatic description, like tiredness, pain, etc., may find it difficult to receive financial compensation from the Swedish sick leave insurance system. One study from our group has revealed that although a very high percentage of MS patients showed no sign of neurological dysfunction when physically examined, in fact, almost 25%, suffered from pathological tiredness – fatigue – as the only subjective symptom [41].

### Limitations

A limitation of the study is associated with the chosen design, no causal explanations and no follow up. The sample included 323 individuals why self-assessment questionnaires were used. Thus, data rely on self-assessments and on the subjective experiences of the participants. The use of a generic instrument to investigate HRQoL (SF36) can be criticized since it does not cover specific MS-symptoms. However, the SF36 is a well known instrument and widely used in other studies, why the results may be easier to interpret and can also be compared with other groups in the future.

## Conclusions

Work capacity and HRQoL among individuals diagnosed with MS are highly influenced by fatigue which, thus, can be considered as a key symptom. Work capacity was also influenced by heat-sensitivity, cognitive difficulties and emotional distress and significant predictive factors besides fatigue were physical disability, age, sex, and educational level. Remaining at work also gave a better HRQoL.

## Competing interests

The authors declare that they have no competing interests.

## Authors’ contributions

All authors contributed to the planning of the study. GF collected all data and together with A-CE conducted the statistical analyses. In collaboration, GF, A-CE and A-ML wrote the drafts which have been repeatedly discussed and revised by all of the authors. Finally, all of the authors have read and approved the manuscript before submission.

## Pre-publication history

The pre-publication history for this paper can be accessed here:

http://www.biomedcentral.com/1471-2458/13/224/prepub
